# Exploring Salt Reduction Strategies: Navigating Policy Implementation in the Middle East

**DOI:** 10.1002/fsn3.71023

**Published:** 2026-04-19

**Authors:** Fatima al Zahra Yakti, Fathima Sahar Faisal, Juman Ali Yaghi, Lana Basel Abusalah, Syed Zamzam, Ayoub Al‐Jawaldeh, Reema Tayyem, Tahra ElObeid

**Affiliations:** ^1^ Department of Nutrition Sciences, College of Health Sciences QU Health, Qatar University Doha Qatar; ^2^ Regional Office for the Eastern Mediterranean (EMRO) World Health Organization (WHO) Cairo Egypt

**Keywords:** consumer education, Middle East, public health, salt initiatives, salt intake, stakeholder engagement

## Abstract

Cardiovascular disease (CVD) remains the leading cause of mortality worldwide. The World Health Organization (WHO) estimates that reducing daily salt intake by 5 g could lower global CVD rates by up to 17%. In the Middle East, excessive salt consumption poses a critical public health challenge, contributing significantly to CVD and related conditions. This review examines salt reduction efforts across the region, focusing on policy interventions, implementation strategies, and barriers. Common initiatives include reformulation of staple foods, consumer education campaigns, and front‐of‐pack labeling (FOPL). However, progress is constrained by limited intake data, funding gaps, reliance on imports with unregulated salt content, and insufficient cross‐sector collaboration. Additional obstacles include socioeconomic disparities, political instability, workforce shortages, and industry resistance. To address these challenges, countries have adopted strategies involving stakeholder engagement, monitoring and evaluation systems, and consumer awareness programs. Evidence from selected case studies demonstrates the importance of integrating cost‐effectiveness considerations into policy design, particularly in resource‐constrained health systems. Overall, sustainable salt reduction in the Middle East requires stronger multi‐sectoral collaboration, robust surveillance mechanisms, and region‐specific adaptations to overcome unique social, economic, and political barriers.

## Graphical abstract

Cardiovascular disease (CVD) is the leading global cause of death, and reducing daily salt intake by 5 g could potentially lower global CVD rates by 17%, according to the WHO. In the Middle East, high salt consumption presents major health challenges, with efforts like food reformulation, consumer education, and labelling facing obstacles such as limited sodium intake data, funding issues, and political instability. Effective salt reduction strategies in the region require multi‐stakeholder collaboration, improved awareness initiatives, and economically viable policies to reduce pressure on healthcare systems and promote long‐term public health improvements.

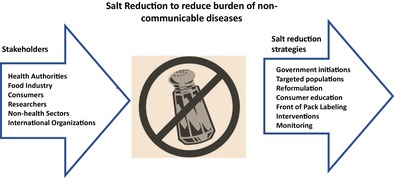

## 1 Introduction

Cardiovascular diseases (CVD) stand at the forefront of the primary cause of worldwide mortality, resulting in 17.9 million deaths each year, constituting 31% of all global fatalities (World Health Organization 2013a). The primary risk factor for CVD is hypertension (Al‐Jawaldeh et al. 2021), and consuming too much sodium is proven to significantly increase the risk of both hypertension and CVD‐related death (He et al. 2013). According to recent statistics from the Global Burden of Disease research, 70 million disability‐adjusted life years (DALYs) and 3.2 million deaths were caused by increased salt intake in 2017, while 10.4 million deaths and 218 million DALYs were caused by hypertension (Stanaway et al. 2018). The World Health Organization (WHO), seeing the urgent need for action, has approved reducing salt and/or sodium intake as a high priority, considering it to be one of the most economical and feasible strategies to mitigate the risk of CVD, coronary heart disease (CHD), and stroke (World Health Organization 2014). It implies that a 5 g daily salt reduction could potentially result in a 17% global drop in CVD rates (World Health Organization 2010). The idea that population‐level sodium consumption reduction is a workable public health strategy is supported by compelling data (Trieu et al. 2015). Urinary sodium excretion and the risk of CVD mortality are positively correlated, according to a recent systematic analysis of prospective studies. This relationship is especially evident when sodium intake is greater than 2.4 g/day (Milajerdi et al. 2019). The World Health Assembly, held in 2013, saw the unanimity of WHO Member States in endorsing the bold global objective of attaining a 30% relative decrease in the mean population's sodium intake by 2025, in comparison to a baseline of 2010 (Al‐Jawaldeh et al. 2021). Objective 3.4, which calls for a decrease in early NCD‐related deaths, is expected to be significantly closer to reality if the global sodium reduction objective is met.

Several countries in the Middle East have developed different policies and strategies to reduce salt. Iran has implemented a comprehensive salt reduction strategy which extends to various food categories (Al‐Jawaldeh, Hammerich, et al. 2020). The acceptable salt levels have been lowered for snacks (from 2.5% to 1.5%), bread (from 2.3% to 1.8%), potato chips (from 1.5% to 1%), and canned tomato paste (from 3% to 2%) (Al‐Jawaldeh, Hammerich, et al. 2020). In 2013, a Salt and Fat Intake Reduction Task Force established in Kuwait developed voluntary agreements to steadily reduce the amount of salt in bread and cheese. In a similar vein, the Kingdom of Saudi Arabia (KSA) established a voluntary reformulation effort involving local and international food enterprises and importers, as well as rules supporting voluntary reformulation to lower salt levels (Al‐Jawaldeh, Hammerich, et al. 2020). KSA imposed a 1% compulsory upper limit on salt content in bread in 2019 (Al Jawaldeh and Al‐Khamaiseh 2018). With a benchmark aim of 0.5%, Oman and the United Arab Emirates (UAE) have assessed food standards and mandated maximum salt levels in bread (Al Jawaldeh and Al‐Khamaiseh 2018). Kuwait and Qatar have successfully reduced salt levels in bread by at least 20% since 2013 through collaboration with the primary supplier (Al Jawaldeh and Al‐Khamaiseh 2018). Jordan and Bahrain have formulated legislation and established targets for salt reduction in bread. Additionally, in Palestine, cooperation with bakeries commenced in 2016, leading to the establishment of an upper limit for salt content in bread at 0.9%. Therefore, our aim in the present review was to summarize the current salt reduction policies in Middle Eastern countries. This review begins with a detailed methodology outlining the search strategy, databases, and inclusion criteria employed to gather evidence on salt reduction policies in the Middle East. It then moves to an analysis of sodium intake and dietary patterns, establishing the context for understanding the region's nutritional challenges. Building on this, the discussion of cardiovascular and other health consequences underscores the urgency of effective interventions. The subsequent examination of implementation approaches highlights the diverse strategies governments have adopted, followed by an exploration of the economic considerations that determine their feasibility and sustainability. Attention then shifts to the obstacles—social, political, and logistical—that may hinder progress, before presenting a comprehensive overview of national initiatives across Middle Eastern countries. Monitoring and evaluation mechanisms are described next, linking policy implementation with accountability and impact assessment. The narrative then delves into to research priorities and program evaluation, emphasizing the need for robust data and frameworks to guide future efforts. Future directions are proposed to enhance regional collaboration, innovation, and effectiveness of salt reduction policies, culminating in a conclusion that synthesizes insights and provides actionable recommendations for policymakers and public health stakeholders.

## 2 Methodology

PubMed, Cochrane, and Google Scholar databases were searched for different salt reduction policies available in different countries within the Middle East region. Although this review adopts a narrative approach, steps were taken to minimize potential bias. Independent literature searches were conducted by multiple authors across major databases. A comprehensive search was conducted for relevant articles published in English between 2010 and 2024. The search approach involved using the following terms to identify exposures to Salt reduction OR Sodium reduction OR salt reduction policies OR sodium reduction policies OR salt policy OR sodium policy OR reducing salt OR reducing sodium OR reducing salt policy OR reducing sodium policy OR salt reduction strategies OR salt and Middle East OR Iran OR Bahrain OR Egypt OR Iraq OR Iran OR Jordan OR Kuwait OR Lebanon OR Oman OR Palestine OR Qatar OR Syria OR Saudi Arabia OR Turkey OR United Arab Emirates OR Yemen OR Arab countries OR GCC AND/OR health impact OR health OR CVD AND/OR economic implications OR cost effectiveness OR healthcare expenditure OR productivity loss OR hospitalization cost AND/OR stakeholder OR stakeholder. In addition, findings were triangulated with authoritative sources such as World Health Organization (WHO) regional reports and governmental policy documents. This multi‐step process aimed to reduce the risk of overlooking relevant studies and to enhance the reliability and transparency of the evidence synthesis. Table 1 outlines the search strategy in detail.

**TABLE 1:** Literature search strategy.

List	Details
Search date	April–May 2024
Databases	PubMed, Cochrane, and Google Scholar
Search terms	“Salt reduction” “Sodium reduction” “salt reduction policies” “sodium reduction policies” “salt policy” “sodium policy” “reducing salt” “reducing sodium” “reducing salt policy” “reducing sodium policy” “salt reduction strategies” “salt” AND/OR “Middle East” “Arab countries” “Bahrain” “Egypt” “United Arab Emirates” “Iraq” “Iran” “Jordan” “Syria” “Kuwait” “Lebanon” “Palestine” “Qatar” “Saudi Arabia” “Turkey” “Yemen” “Oman” “GCC” AND/OR “health impact and salt” “salt and health” “CVD” AND/OR “economic implications” “cost effectiveness” “healthcare expenditure” “productivity loss” “hospitalization cost” AND/OR “Stakeholder” “Stakeholder engagement”
Time frame	2010–2024
Inclusion/exclusion criteria	All study designs except for narrative/literature review. Only English Studies were included
Collection procedure	Literature search conducted independently by all authors.

## 3 Salt Reduction Policies Among Twelve Middle East Countries

### 3.1 Salt Reduction Policy in Iran

Iran was among the first Middle Eastern countries to apply a comprehensive strategy to reduce the intake of salt. The initiative, spearheaded by the government, implemented mandatory regulations to control salt levels across various food products (Al‐Jawaldeh et al. 2021). In 2015, maximum salt thresholds were established for normally consumed canned foods, including tomato paste and salty snacks, as well as all varieties of bread, initially set at 1.8%. Subsequent years saw a progressive reduction, with the salt content in bread lowered to 1% by 2016–2017 (Al‐Jawaldeh et al. 2021). Similarly, salt standards were revised downwards for cheese and dough (a fermented drink), from 4% to 3% and from 1% to 0.8%, respectively (Al‐Jawaldeh et al. 2021). Additionally, in 2016, the government took a proactive step by introducing mandatory nutritional traffic light labeling for all imported and domestic packaged foods, excluding certain unprocessed or non‐formulated items. In 2018, the use of salt was prohibited in probiotic yogurts, in addition to public education campaigns via television advocating for lower salt consumption (Al‐Jawaldeh et al. 2021).

### 3.2 Palestine

The National Health Strategy for 2021–2023, introduced by the government in 2019 under the Ministry of Public Health (MOPH) leadership, aims to reformulate food categories with a focus on gradually cutting down salt content in bread, following a mandatory approach. Specific targets have been set for each year: 0.9 g/100 g in 2019, 0.8 g/100 g in 2021, 0.7 g/100 g in 2022, and 0.6 g/100 g in 2023 (Al‐Jawaldeh et al. 2021).

### 3.3 Jordan

In 2019, the government‐led initiative to reduce salt intake focused specifically on Arabic bread. This mandatory action, undertaken in collaboration with bakeries, aimed to ensure that Arabic bread contained less than 1% of dry weight salt. Additionally, existing legislation was revised by the government to establish benchmarks for salt content in highly consumed foods, including cheeses. Furthermore, various strategies such as social marketing campaigns and dietary guideline promotions were used to raise awareness and encourage healthier dietary habits (Al‐Jawaldeh et al. 2021).

### 3.4 Egypt

The government came up with a plan in 2017 that includes requiring ministerial decrees to lower the salt content by 30% in Baladi Bread that was subsidized by the government. Additional measures included offering bakeries optional training on how to reduce salt in bread by 20%. Additionally, awareness campaigns were created to encourage customers to cut back on their salt intake by raising their understanding of the risks associated with excessive salt consumption (Al‐Jawaldeh et al. 2021).

### 3.5 Syria

One of the main countries that implemented and tested the salt reduction policy was Syria. The rise in CHD mortality in Syria can mainly be attributed to higher levels of population systolic blood pressure (SBP). Over the period from 1996 to 2006, approximately 2700 CHD fatalities have been linked to a 4.5% and 9.5% rise in SBP among Syrian men and women aged 25 and above, respectively (Rastam et al. 2012; Wilcox et al. 2015). Considering salt intake patterns in the Eastern Mediterranean region (EMR) and the effectiveness of prior salt reduction initiatives in different contexts (Appel et al. 2012; Cappuccio et al. 2011; Rastam et al. 2012), three potential policies were assessed:

1. A nationwide health promotion campaign aimed at urging individuals to cut down on their salt intake (Appel et al. 2012; Cappuccio et al. 2011; Rastam et al. 2012).
2. A requirement for manufacturers to label prepackaged foods with the amount of salt in order to provide consumers with low‐salt options; and (Appel et al. 2012; Cappuccio et al. 2011; Rastam et al. 2012).
3. A mandatory reformulation program requiring manufacturers to reduce the amount of salt in prepackaged foods. Healthcare expenses were reduced by 4.5% when all three policies were implemented. Based on the most accurate estimations, the health promotion campaign, labelling, and the combination of all three measures were all found to be cost‐effective. A comprehensive strategy that includes required salt content reformulation in manufactured foods, mandatory food labelling on packaged goods, and a statewide health promotion campaign is the most promising method for lowering the prevalence of CHD in Syria (Appel et al. 2012; Cappuccio et al. 2011; Rastam et al. 2012).

### 3.6 Lebanon

One of the first nations to create regulations for salt reduction was Lebanon. The government works with academia to lead the Lebanese Action on Sodium and Health (LASH) initiative, which was founded in 2012. It is voluntary in nature and focuses on evaluating salt intake and sources in addition to raising public awareness of salt. Working along with the Ministries of Industry and Health, LASH aims to create national guidelines for bread salt content. Furthermore, LASH intends to work with a communications firm to initiate a nationwide campaign to lower salt intake, to inform people about the dangers of consuming too much salt and enabling them to choose healthier foods (Al‐Jawaldeh et al. 2021).

### 3.7 Bahrain

It was shown that CVDs constitute 26% of total deaths in Bahrain, according to the WHO. The Nutrition Section of the Ministry of Public Health (MOPH) is primarily responsible for initiatives aimed at reducing salt intake (Alhamad et al. 2015). A pilot study, conducted in collaboration with the Eastern Mediterranean Regional Office (EMRO), evaluated bread's salinity. Twenty samples were taken from different bakeries in Bahrain, and the results showed that the salt concentration was far greater than what the WHO suggested. Pending clearance from the MOPH, the Nutrition Section has suggested an action plan for a yearly 10% reduction in the amount of salt in bread (Alhamad et al. 2015).

A ministerial order aimed at forming a multisectoral group to lower the salt content in bread goods was released in 2014. The committee's goals are to create a plan of action and strategy for reducing the amount of salt in baked goods, enforcing food labeling laws that disclose salt content, and creating laws that have monitoring systems in place for when they are put into effect (Alhamad et al. 2015). Additionally, the MOPH spearheaded a government program in 2018 that mandated a mandatory decrease in salt in Arabic bread, aiming for a 20% annual decrease in salt content over five years until reaching the targeted 0.5% salt level based on dry flour weight (Al‐Jawaldeh et al. 2021). Additionally, nutrition labels on baked goods are required to clearly indicate the quantity of added salt (Al‐Jawaldeh et al. 2021).

### 3.8 Kuwait

According to the WHO, CVDs constitute 41% of total deaths in Kuwait. The Food and Nutrition Administration (FNA), part of the MOPH, is the primary governmental body responsible for overseeing salt reduction activities (Nawal Alhamad et al. 2015). Studies have revealed that the primary food sources of salt in the Kuwaiti diet were foods prepared at home during food preparation. Bread consumption emerged as the second major source of salt intake.

The Kuwait Flour Mills and Bakeries Company (KFMBC) is the primary producer of bread in Kuwait, accounting for 80% of total bread production. In partnership with KFMBC, MOPH decided to implement WHO recommendations for reducing salt intake. A 10% reduction in salt content in bread was achieved in March 2013; then, in August of the same year, a further 10% cut that took place six months later. By October 2013, the company had reduced the salt content of almost all of their bread varieties by 20%. The amount of salt in bread is regularly checked (Nawal Alhamad et al. 2015).

Over 95% of the food consumed in Kuwait is imported, indicating a significant reliance on foreign food. Meat, cheese, chips, and breakfast cereals are examples of processed foods that greatly increase the amount of salt consumed in Kuwaiti diets. A strategy was developed to promote collaborations and include food corporations in health‐related initiatives. Introducing solutions for reducing salt intake and working with the private sector to create a workable and efficient action plan for reducing salt intake gradually were the key goals. A comparable strategy with the same objectives was used with the largest restaurant franchise operators in Kuwait (Nawal Alhamad et al. 2015).

Additionally, salt standards for cheese were revised. In 2017, a government‐led strategy aimed to promote nutrition in school children mandated a salt content target of ≤ 1.5 g/100 g for corn and potato crisps (Al‐Jawaldeh et al. 2021). More recently, in 2021, the government, through the FNA in cooperation with the Patient Helping Fund Society (PHFS), enforced mandatory measures, including the application of the traffic light system on food items in governmental hospitals cafeterias and canteens, and revising hospital menus to lower salt levels (Al‐Jawaldeh et al. 2021).

### 3.9 Oman

According to the WHO, 33% of all fatalities in Oman are caused by CVDs. The nation's efforts to reduce salt consumption are supervised by the Ministry of Public Health (MOPH). The National Nutrition Survey estimates that the average daily intake of salt is between 11 and 12 g. Plans are underway to bring salt intake down to WHO guidelines, with an emphasis on high‐salt goods such as processed meats, cheese, and bread that are frequently consumed (Nawal Alhamad et al. 2015).

The Omani Standard for Bread, introduced in 2019 under the leadership of the Ministry of Commerce, Industry, and Investment Promotion (MOCI), aimed to reduce salt content in bread. Initially voluntary, a 10% reduction was implemented in late 2015 across major bakeries, followed by an additional 10% reduction to a total of 20% (Al‐Jawaldeh et al. 2021; Nawal Alhamad et al. 2015). This reduction became mandatory in May 2019, with flat bread (Arabic bread) required to contain 0.5% salt and other varieties limited to 1%. Additionally, a planned 30% reduction in salt content for food products was proposed. Another upcoming initiative, scheduled for 2021–2022 and led by the government and NGOs, involves social marketing campaigns, TV advertising, and events to promote salt consumption reduction (Al‐Jawaldeh et al. 2021; Nawal Alhamad et al. 2015).

### 3.10 Qatar

In Qatar, 24% of deaths are related to CVDs. Activities in Qatar aimed at reducing salt are supervised by the Supreme Council of Health (SCH). Bread and other baked goods were found to be the primary sources of salt in the diet in a nationwide study conducted by the SCH. Plans exist to lower the amount of salt in bread by 30%. The primary bread manufacturer, Mesaieed Bakery (Qbake), has tested samples to see if the salt content has decreased (Nawal Alhamad et al. 2015).

The WHO Salt Reduction in Bread Initiative, launched in 2013 under the leadership of the Ministry of Public Health (MoPH) as part of the Nutrition and Physical Activity Action Plan 2011–2016, aims to achieve a 20% reduction in salt content in bread (Al‐Jawaldeh et al. 2021; Alhamad et al. 2015). This reduction has been initiated in major national bakeries and is mandatory for bread samples containing more than 0.8% salt. Social marketing campaigns, such as Salt Awareness Week, are conducted annually by the MoPH to raise awareness through various channels including TV advertising, interviews, and events (Nawal Alhamad et al. 2015). Additionally, ongoing efforts led by the MoPH involve implementing food and beverage guidelines, school canteen regulations, and educational sessions in various settings to promote healthier dietary habits among the population (Al‐Jawaldeh et al. 2021; Nawal Alhamad et al. 2015).

### 3.11 Kingdom of Saudi Arabia (KSA)

It was reported that CVDs in KSA account for 46% of total deaths. The Ministry of Public Health (MOPH) oversees salt reduction activities in the country. The goal of the early stages of salt reduction efforts is to lower the amount of salt in bread. The MOPH wants to enact a directive reducing salt consumption by 10% each year until a 30% reduction is attained. One of the challenges is lowering the salt content in traditional breads, which are frequently made based on the tastes of individual bakers (Nawal Alhamad et al. 2015).

Aiming to minimize salt content in a variety of food categories, the Saudi Food and Drug Authority (SFDA) is in charge of the government's Healthy Food Strategy, which is a component of Vision 2030 and was introduced by the government in 2018 (Al‐Jawaldeh et al. 2021; Nawal Alhamad et al. 2015). Focusing on a large range of foods, such as breads, ready meals, cheeses, butter, margarines, biscuits, cakes, biscuits, and salty snacks, it also includes soups and sauces. The strategy aims to achieve specific salt limits, such as 1.0 g/100 g for all types of bread and 1 g/100 mL for yogurt drinks (Al‐Jawaldeh et al. 2021; Nawal Alhamad et al. 2015). In tandem with these regulatory efforts, social marketing campaigns, events, and social media posts have been used to raise awareness and encourage healthier choices among consumers. Moreover, the initiative emphasizes the importance of informed decision‐making through voluntary traffic light labeling, which categorizes foods based on their salt content, and extends its reach into various settings including workplaces, schools, and hospitals (Al‐Jawaldeh et al. 2021; Nawal Alhamad et al. 2015).

### 3.12 United Arab Emirates (UAE)

In the UAE, 30% of all deaths are related to CVDs. Activities in the UAE aimed at reducing salt are supervised by the MOPH. Identifying the primary bread producers and reviewing their production, distribution, and usage of standardized recipes are the first stages in putting a salt intake reduction strategy into practice. The idea is to gradually reduce the amount of salt in bread (Nawal Alhamad et al. 2015).

Under the direction of the Ministry of Health and Prevention (MOPHAP), the government‐led Reformulate Food Products program has been a part of the National Action Plan since 2017. It promotes the voluntary reformulation of bread and other food products to contain less than 0.5% salt (Al‐Jawaldeh et al. 2021; Nawal Alhamad et al. 2015). The Reduction of Salt Intake by 30% National Action Plan, another 2017 endeavor, suggests media campaigns and activities to increase public and food industry awareness. In 2019, initiatives like “Exclude Salt from Your Food Menu” and “Pay Attention to the Dangers of Excessive Salt Intake!” utilized Instagram to promote low‐salt food choices and offer preparation tips (Al‐Jawaldeh et al. 2021; Nawal Alhamad et al. 2015). Additionally, planned initiatives for 2020 include voluntary traffic light labeling, to be made mandatory by 2022, aiming to provide clearer guidance on salt content in foods (Nawal Alhamad et al. 2015).

Jordan have reported sodium reductions of 10%–15% in bread; however, loaves still contain an average of 400–500 mg sodium per 100 g, which remains well above the WHO recommended limit (Nasreddine et al. 2014). Saudi Arabia's mandatory reformulation of bread demonstrated the potential of regulatory measures, while voluntary agreements in Turkey achieved only modest reductions of 8%–12% in processed meats such as sausages and cold cuts, which often contain 1500–2000 mg sodium per portion (Al Jawaldeh et al. 2018). Packaged soup mixes, which can exceed 1800 mg sodium per serving, were reformulated in Saudi Arabia with a 10% cut, showing that modest changes in high‐sodium products can yield substantial public health gains without compromising sales. Similarly, salty snacks such as chips and crackers, typically containing 700–1000 mg sodium per 100 g, were reduced by 15% in some GCC countries, accompanied by front‐of‐pack labelling to guide consumer choices (Musaiger et al. 2011). Taken together, these examples illustrate the breadth of targeted products but also underscore a critical limitation: percentage reductions alone may leave foods substantially above safe intake thresholds. Hence, strategies that integrate mandatory reformulation, clear sodium benchmarks, and consumer‐facing labelling appear more effective than voluntary or awareness‐based approaches in driving sustained population‐wide reductions.

Cross‐country synthesis. The emerging pattern is clear; mandatory, multi‐category reformulation plus mandatory interpretive labeling, anchored in multisector governance and delivered through institutional settings (schools, hospitals, workplaces), consistently outperforms voluntary or education‐only approaches. Countries that coupled bread standards with broader category limits and labeling (e.g., Iran; Bahrain; Oman's later phase; Saudi Arabia's multi‐category limits) exhibit the most credible pathway to population‐level impact (Al‐Jawaldeh et al. 2021; Nawal Alhamad et al. 2015). Where strategies remain voluntary or narrowly focused (e.g., Lebanon; early‐stage UAE), uptake is more variable and effects more limited. Modeled evidence from Syria reinforces that comprehensive packages (reformulation + labeling + promotion) are not only more effective but can be cost‐saving, strengthening the policy case for mandates and coordinated implementation across the region (Appel et al. 2012; Cappuccio et al. 2011; Rastam et al. 2012; Wilcox et al. 2015).

## 4 Consequences of Excess Salt Intake on Health

Excessive intake of salt has a notable impact on human health, as it is linked to several health hazards. High sodium consumption is linked with elevated blood pressure, which ultimately contributes to cardiovascular morbidity and mortality, resulting in approximately 5 million deaths annually worldwide (Hunter et al. 2022). Intake of excess salt is associated with many conditions including hypertension, CVD, osteoporosis, kidney disease, stomach cancer, etc. (Better Health Channel 2022). Elevated intake of sodium represents a significant risk factor for hypertension, which is a precursor to CVD and stroke. Research indicates a robust positive correlation between sodium consumption and systolic blood pressure, highlighting that decreased intake of sodium can effectively lower blood pressure levels (Farquhar et al. 2015). The American Heart Association stresses the significance of monitoring sodium intake to uphold optimal heart health. They advise on reducing dietary sodium by implementing strategies such as lowering the sodium content of foods and transitioning to low‐sodium alternatives. These approaches are aimed at fostering improved cardiovascular health (American Heart Association 2022). The increased risk of chronic kidney disease associated with elevated levels of sodium intake is likely attributable to the kidneys' diminished capacity to expel excess sodium (Farquhar et al. 2015). The risk of osteoporosis is because high salt consumption can result in increased calcium excretion through urine, potentially leading to calcium depletion from bones and raising the likelihood of osteoporosis. Also, according to research findings, an augmented risk of stomach cancer has been associated with a higher intake of salt, sodium, or salty foods (Harvard T.H. Chan School of Public Health 2023).

Diets prevalent in the Middle East region are characterized by notably high salt content, with an average daily intake of sodium ranging from 3.74 to 4.12 g per day, which is equivalent to 9.35–10.3 g of salt. This exceeds the WHO‐recommended limit of 5 g per day by almost double. Certain countries in the region, such as Turkey, exhibit even higher average salt intakes, reaching up to 15 g per day (Arici 2016). A study conducted in Jordan indicated an average daily sodium intake of 179 mmol (4.1 g), with males showing a higher intake at 186 mmol (4.3 g) compared to females at 173 mmol (4.0 g) (Alawwa et al. 2018). In the Middle East, hypertension stands as the primary contributor to CVD mortality, rendering the region a “hotspot” for CVDs. This designation is largely attributed to the profound impact of elevated sodium consumption prevalent in the area. The primary dietary sources of sodium in the Middle East encompass bread, dairy products, processed meats, and condiments/spices (Hanbazaza and Mumena 2020). For example, in 2015, bread was identified as the primary source of dietary salt for individuals in Gulf Cooperation Council (GCC) countries like the UAE, Qatar, KSA, Oman, Kuwait, and Bahrain (Alhamad et al. 2015). In Lebanon, the leading sources of dietary sodium were identified as bread (25%), processed meat (12%), and dairy products such as cheese and yogurt (10%) (Nasreddine et al. 2014).

Efforts aimed at reducing salt intake in EMR are paramount, given the importance of addressing noncommunicable diseases (NCDs), notably CVDs. Initiatives focusing on implementing strategies to decrease salt consumption play a pivotal role in preventing such diseases (Alkhunaizi et al. 2013). Several countries in the region are actively pursuing national salt reduction initiatives. One notable approach involves gradually reducing the salt content of staple foods like bread and setting targets to restrict salt in other food items. For instance, Kuwait has successfully decreased the salt content of bread by 20% through a phased approach, marking a significant milestone in public health advancement (Jawaldeh 2016). Another example in Oman states that they reviewed the food standards and set a benchmark for salt content in bread at 0.5 g/100 g. This provides insights into the potential impact of these policies on public health outcomes and healthcare expenditure (Ayoub Al Jawaldeh et al. 2018).

## 5 Approaches to Salt Reduction Implementation Strategies

Thirteen Middle Eastern countries have implemented comprehensive salt reduction initiatives, utilizing a blend of two or more implementation strategies. Taxation emerges as the least commonly utilized strategy in the region, with only Qatar slated to implement taxation on high salt products, while the UAE is deliberating taxation on unhealthy salty foods as part of their National Action Plan of 2017 (though not yet adopted) (GIFNA (The Global Database on the Implementation of Food and Nutrition Action) 2017). The most prevalent salt reduction initiative is reformulation, implemented by all 13 countries (100%), consumer education in 10 out of 13 countries (77%), initiatives in specific settings in 7 out of 13 countries (54%), and front‐of‐package labelling (FOPL). In EMR, the front‐of‐pack labelling was implemented in high income: Bahrain, UAE, and in middle income: Iran, Morocco, KSA, Tunisia, which accounts for 6 out of 13 countries (46%) (Al‐Jawaldeh et al. 2021). The detailed information is given in Table 2.

**TABLE 2:** Salt reduction strategies initiated by Middle East countries.

Strategies	Action plan
Government led initiatives	Except for Lebanon, where academia collaborated with the government, all identified strategies for salt reduction were primarily spearheaded by governmental authorities.
Targets for population salt intake	Apart from Lebanon and Palestine, 11 countries (85%) have set targets for population salt intake. Bahrain, Jordan, KSA, Kuwait, Oman, and Qatar align with the WHO recommendation of 5 g of salt per day. Egypt, Iran, and the UAE aim for a 30% relative reduction in salt intake. These strategies are incorporated into broader initiatives addressing NCDs or advocating for healthy diets and lifestyles region wide.
Reformulation	Except for Lebanon, governmental authorities lead reformulation initiatives across various countries in Middle East, collaborating with the food industry. Bread is the most commonly targeted food product for reformulation, with eight countries mandating these initiatives. Some countries have extended reformulation efforts to include additional food products like cheese, salty snacks, and canned foods.
Consumer education	Most countries in Middle East, except Palestine, Kuwait and Bahrain, have incorporated consumer education campaigns into their salt reduction strategies. These initiatives, predominantly led by governmental authorities, focused primarily on salt awareness, although some also addressed broader aspects of healthy diets. Additionally, consumer education efforts were often integrated with other salt reduction initiatives across the region.
Front of pack labelling (FOPL)	Five countries in Middle East, comprising KSA, UAE, Iran, Morocco, Tunisia, have implemented FOPL initiatives related to salt. Iran employs a mandatory traffic light labelling scheme for packaged foods, while KSA and the UAE have voluntary traffic light labelling with plans for mandatory adoption in the UAE by 2022. Bahrain focuses on mandatory labelling of baked products specifying added salt.
Interventions in specific settings	Five countries (23%) in Middle East are executing salt reduction initiatives within specific settings, including Qatar, KSA, UAE, Kuwait and Egypt. These initiatives encompass health education programs integrated with salt reduction components, voluntary procurement policies to limit high salt foods availability, hospital menu modifications, food labelling in governmental institutions, and promotion of low‐salt foods in school lunch programs. Planned initiatives in Oman involve establishing supportive environments by providing low‐salt foods in public places and workplaces, while also restricting the availability of high‐salt foods in schools.
Monitoring	Several Middle East countries, including Qatar, Oman, Kuwait, Egypt, Jordan, Iran and the UAE, have established multisectoral national committees to oversee the implementation of salt reduction strategies. Monitoring activities in Kuwait revealed challenges in achieving salt reduction targets in bread and crisp products. Similar monitoring efforts in countries such as Bahrain, KSA, and Qatar are ongoing to ensure compliance with salt reduction policies and to assess the impact of these initiatives on salt content in bread and consumer perceptions.

Despite these initiatives, Middle East countries have encountered various challenges while implementing salt reduction strategies. Firstly, a significant obstacle is the lack of comprehensive data on sodium intake levels in the region, hindering the ability to effectively target and monitor the impact of salt reduction policies. Moreover, insufficient funding and technical capacity pose barriers to the implementation of successful salt reduction programs, particularly in middle‐ and low‐income countries. Additionally, changes in leadership can disrupt the continuity and sustainability of salt reduction initiatives, further complicating progress in this area. Furthermore, the limited engagement of youth, civil society and the media represents a challenge, as their involvement is crucial for building awareness and advocacy efforts around salt reduction. Lastly, inadequate collaboration with ministries is identified as a critical issue, particularly in reducing the amount of salt used in traditional bread and other foods, highlighting the importance of establishing partnerships (Jawaldeh 2016; Al‐Jawaldeh et al. 2021; Al‐Jawaldeh, Hammerich, et al. 2020).

## 6 Stakeholder Engagement

Stakeholders are individuals, groups, or organizations with an interest or concern for a certain policy, issue, or decision (Lemke and Harris‐Wai 2015). These stakeholders may have differing interests, concerns, and perspectives. Hence, engaging them in the policy‐making process ensures that a wide range of viewpoints is considered (Lemke and Harris‐Wai 2015).

For the salt reduction policy to be successfully implemented, it is important to have an effective engagement of stakeholders (Rosewarne et al. 2021). A systematic approach, such as stakeholder mapping, helps identify and involve relevant parties effectively. Stakeholders are categorized based on their interest and influence on the policy. High‐interest, low‐influence groups need regular updates and support. Those with high interest and influence should be consulted for strategic advice, while efforts are made to convince high‐influence, low‐interest groups. Government involvement is essential from the start, especially in health ministries. Engaging the food industry early, communicating benefits, and addressing technical concerns are vital. Collaboration with health professionals, academics, and NGOs is also crucial for wide‐ranging support and public awareness (“Stakeholder Mapping and Engagement”). The stakeholders, which vary from country to country, as reported by the WHO SEARO Salt reduction toolkit usually include (“Stakeholder Mapping and Engagement”):

1. Government ministries such as Health, Trade, Industry, Finance, and Consumer Affairs (World Health Organization 2024; Nawal Alhamad et al. 2015; Almedawar et al. 2015; Sarrafzadegan 2023; “The Hasemite Kingdom of Jordan—National Nutrition Strategy 2023/2030”).
2. Local authorities (World Health Organization 2024).
3. Non‐governmental organizations (NGOs) focusing on health and food‐related issues (World Health Organization 2024; Sarrafzadegan 2023; Almedawar et al. 2015; Nawal Alhamad et al. 2015).
4. Healthcare professionals like doctors, nurses, dietitians, and nutritionists, as well as their representative associations (World Health Organization 2024; Nawal Alhamad et al. 2015; Almedawar et al. 2015; Sarrafzadegan 2023).
5. The food industry, encompassing individual food companies (e.g., soy sauce manufacturers), industry associations, trade bodies, importers, distributors, and wholesalers (World Health Organization 2024; Almedawar et al. 2015; Nawal Alhamad et al. 2015).
6. The Out of Home sector, including chef associations, restaurant associations, street food vendor representatives, high‐profile chefs/street food vendors, distributors, and food aggregators (World Health Organization 2024).
7. Consumer groups and associations (World Health Organization 2024; Sarrafzadegan 2023; Almedawar et al. 2015)

A study done by Alhamad et al. (2015) identified key stakeholders that were involved in the GCC countries in the salt reduction policy. These countries include Bahrain, Kuwait, Oman, Qatar, KSA, and the UAE (Nawal Alhamad et al. 2015).

In Bahrain, stakeholders involved in the endeavor to decrease salt consumption and enhance public health included the MOPH along with its Nutrition Section, as well as the Bahrain Flour Mills Company (BFMC), privately owned bakeries, and a multisectoral committee specifically addressing salt reduction in bread products (Nawal Alhamad et al. 2015). Additionally, collaboration extends to the Eastern Mediterranean Regional Office of the World Health Organization (WHO EMRO), the Bahraini population encompassing adults and children, and health professionals and researchers actively participating in pertinent studies and initiatives (Nawal Alhamad et al. 2015).

Similarly, Kuwait's efforts enlist the MOPH and its FNA, Kuwait Flour Mills and Bakeries Company (KFMBC), private sector food entities, and restaurant franchise operators, alongside health professionals and researchers contributing to related studies (Nawal Alhamad et al. 2015).

In Oman, the MOPH spearheads efforts, supported by the National Nutrition Survey, the Director of Nutrition, and bakeries, while engaging the Omani population and proposed national task force for salt reduction, as well as health professionals and researchers (Nawal Alhamad et al. 2015).

Qatar's strategy involves the Supreme Council of Health, Mesaieed Bakery (Qbake), the Central Food Laboratory, WHO EMRO bakeries, the Qatari population, health professionals, and researchers (Nawal Alhamad et al. 2015).

KSA focuses on collaboration between the MOPH, Saudi Arabian population, bakeries, Gulf Nutrition Committee, and health professionals and researchers (Nawal Alhamad et al. 2015).

The UAE engages the MOPH, UAE population, bread producers and bakeries, private sector entities, health professionals and researchers, and the broader GCC community (Nawal Alhamad et al. 2015).

Additionally, in Lebanon, various stakeholders played crucial roles in the efforts to decrease salt intake and enhance public health. The Ministry of Public Health (MOPH) and the Ministry of Industry (MoI) were actively involved, along with the Syndicate of bakeries. Academic institutions like the American University of Beirut (AUB) and its Vascular Medicine Program contributed significantly (Almedawar et al. 2015). Furthermore, NGOs such as Consumers International in Lebanon and the Lebanese Association for the Study of Hypertension (LASH) played vital roles. These efforts were supported by the Lebanese population, with the WHO EMRO providing valuable guidance and support throughout the initiative (Almedawar et al. 2015).

In Iran, key stakeholders involved in decision‐making, policy implementation, and those affected by salt reduction initiatives were diverse and influential (Sarrafzadegan 2023). Decision‐makers included legislative authorities such as the Iranian parliament's high Council for Health, Food Security, and Nutrition, along with policymakers like the MOPH and the Food and Drug Administration (FDA) (Sarrafzadegan 2023). Executive forces encompassed various ministries, educational institutions, food industries, and unions. End‐users, including different age groups and high‐risk individuals, were the focus of these initiatives (Sarrafzadegan 2023). Partnerships were established with media, educational, and health institutions, alongside religious authorities and celebrities (Sarrafzadegan 2023). However, there was potential opposition from producers of high‐salt processed foods and certain syndicates, such as fast food chains, restaurant owners, and producers (Sarrafzadegan 2023).

Stakeholders involved in Jordan's efforts to reduce salt intake and improve public health included various government entities (“The Hasemite Kingdom of Jordan—National Nutrition Strategy 2023/2030”). These included MOPH, the Jordan Strategy for Health and Medical Development, and the Jordan Food and Drug Administration (JFDA). Additionally, the Ministry of Industry and Trade was also involved in these efforts (“The Hasemite Kingdom of Jordan—National Nutrition Strategy 2023/2030”).

The involvement of stakeholders observed in the Middle East countries is similar to countries in the world as seen in India, Nigeria, UK, or Australia. In India, stakeholders in salt reduction policy included government representatives, civil society members, industry professionals, and consumers, all supporting the need for a comprehensive salt reduction program. Recommendations included consumer awareness, consumer‐friendly food labeling, industry promotion of salt reduction, and increased research on salt consumption (Gupta et al. 2018). In the UK, stakeholders such as the Department of Health, the Food Standards Agency, and Public Health England collaborated with NGOs, the food industry, and public health advocates on initiatives including awareness campaigns and reformulation targets (Hyseni et al. 2017). In Nigeria, regulators, producers, academia, consumers, and healthcare staff collaborated on initiatives like obligatory sodium limits, advertising restrictions, mass‐media campaigns, and adjusted food labeling (Sanuade et al. 2023). In Australia, the Victorian Salt Reduction Partnership, involving various organizations, aimed to reduce salt intake through consumer awareness campaigns, industry engagement, and policy strengthening (Rosewarne et al. 2021).

Hence, different stakeholders in the Middle East and other countries collaborated on the development and implementation of salt reduction policies. Strategies included consumer awareness, industry engagement, labeling improvements, and research enhancement.

## 7 Potential Barriers and Challenges in Salt Reduction Implementation

While the majority of Middle Eastern countries have implemented national policies to reduce salt intake, the population's intake is still higher than WHO's recommended upper limit of 5 g per day, largely due to various challenges and barriers present in the region (Al‐Jawaldeh et al. 2021).

Many of these challenges were identified, such as:

### 7.1 The Influence of Socioeconomic Levels and Food Security

The diversity in socioeconomic levels exists in the area, between the world's highest and lowest income, resulting in health inequity within countries and food insecurity (Al‐Jawaldeh, Hammerich, et al. 2020). Consequently, individuals tend to shift their diets from traditional diets to energy‐dense, processed food and high fast‐food diets. This shift may stem from the affordability of such diets and subsidizing foods that contain high fat and salt, or the influence of higher socioeconomic statuses (“Moving forward on salt and fat reduction in the Region” 2015). These fast‐food diets are characterized by their high fat, high sugar, and high salt content (“Moving forward on salt and fat reduction in the Region” 2015).

In addition, food security was altered due to the recent political and economic crises and armed conflict in the region. This impaired the feasibility of policy implementation and evaluation in many countries in the region, like Syria and Yemen, that are facing a large food crisis (Al‐Jawaldeh, Hammerich, et al. 2020; Wilcox et al. 2015).

In Australia, taxation on unhealthy food high in saturated fat and salt resulted in a significant reduction in sodium and energy intake, accompanied by a decrease in fruits and vegetables intake due to the cross‐price elasticity effect (Cobiac et al. 2017). Conversely, subsidies on fruits and vegetables increased their intake but also led to unintended rises in sodium and energy intake (Cobiac et al. 2017). This study underscores the necessity of implementing a combination of policies to address these issues in the region without adversely affecting any socioeconomic groups.

### 7.2 Multisectoral Action and Nutrition Professionals

While the significance of multisectoral action on nutrition is acknowledged, the involvement of sectors beyond health often remains limited in practice in Middle Eastern countries (Al‐Jawaldeh, Hammerich, et al. 2020). Consequently, there is inadequate enforcement of regulations and laws during policy implementation. Middle Eastern countries generally exhibit a low density of trained nutritionists and dietitians (World Health Organization. Regional Office for the Eastern Mediterranean 2019), impacting the government's capacity to effectively implement and monitor policies (Al‐Jawaldeh, Hammerich, et al. 2020). This deficiency resulted in a lack of available data in the region (“Moving forward on salt and fat reduction in the Region” 2015).

Although Iran is following WHO recommendations to prevent NCDs through nutrition programs and policies, salt intake in Iran remained above the recommended levels of an average of 9.5 g/day (Al‐Jawaldeh et al. 2021). The problem is associated with structures within the Ministry of Public Health where there is no cooperation between sectors and key stakeholders. There is not enough power to either draw corporations or force regulations and rules; these lead to implementation problems (Amerzadeh and Takian 2020).

### 7.3 Trading and Industries

Ensuring the collaboration of importers and manufacturers is crucial in implementing such policies, given their significant influence. However, the reliance on imported food in Middle East countries may pose a barrier to such policies unless specific regulations and guidelines are established for imported products (“Moving forward on salt and fat reduction in the Region” 2015; Al‐Jawaldeh, Hammerich, et al. 2020). Furthermore, industries are actively resisting policymakers' efforts to regulate salt intake through taxes (Al‐Jawaldeh, Hammerich, et al. 2020), reformulate high‐salt food items, such as bread (Al Jawaldeh and Al‐Khamaiseh 2018).

Policymakers in the Middle East face challenges in motivating industries to reformulate food products high in salt (Al Jawaldeh and Al‐Khamaiseh 2018). Bakeries and industries often cite technical limitations as a reason for their resistance, expressing concerns about maintaining the palatability and physical properties of bread and other processed food (Al Jawaldeh and Al‐Khamaiseh 2018). However, Jordan has succeeded in producing flat bread with a lower salt content (4.28 g/kg) compared to other countries in the region (Al Jawaldeh and Al‐Khamaiseh 2018). This success indicates that it is feasible for other countries to do the same without encountering significant concerns.

The UK's salt reduction program stands out as a successful example, achieved through cooperation with food industries to inspire product reformulation (Jawaldeh 2016). This initiative involved engaging various sectors of the food industry. Notably, salt levels in certain food items were decreased by as much as 55%. Since 2003, the average population intake has decreased from 9.5 g/day to 8.1 g/day (Jawaldeh 2016).

Front of food pack labeling (FOPL) has had a huge impact on consumers' consciousness and awareness, prompting healthier food choices. Moreover, it has the potential to incentivize food producers to reformulate products to avoid negative labeling on their packages (Jachimowicz‐Rogowska and Winiarska‐Mieczan 2023). In 2016, in Chile the addition of a black stop sign symbol to foods surpassing limits on sodium, saturated fat, energy, and sugars resulted in a reduction in salt content in processed foods within a year. This indicates that food producers are indeed altering their product compositions in response to Jachimowicz‐Rogowska and Winiarska‐Mieczan (2023).

Several countries in the Middle East have implemented compulsory FOPL such as Iran (Moslemi et al. 2020) and Bahrain (Al‐Jawaldeh et al. 2021). Meanwhile, KSA and UAE have embraced voluntary traffic light labeling, with intentions to transition to mandatory labeling in the near future (Bin Sunaid et al. 2021; Al‐Jawaldeh, Rayner, et al. 2020).

### 7.4 Population Knowledge and Awareness

Studies conducted to evaluate consumer awareness in Eastern Mediterranean countries revealed a lack of knowledge regarding the detrimental health effects associated with excessive salt consumption. Furthermore, participants exhibited limited comprehension of food labeling (Al‐Jawaldeh et al. 2021).

Existing research on attitudes toward salt within the region is limited. Findings suggest that only a small percentage of individuals actively seek information about salt content on food labels. Additionally, a considerable portion of the population habitually adds salt to homemade meals or dishes served at the table (Al‐Jawaldeh et al. 2021).

It is recommended that each country within the region reassess its consumer education strategies and adapt them to effectively target the specific gaps in knowledge and attitudes regarding salt intake among their populations. As previously highlighted, front‐of‐package labeling has proven to be one of the most efficacious initiatives globally. Therefore, it is recommended for all countries in the region to mandate its implementation to enhance consumer awareness and promote healthier dietary choices.

In a controlled intervention study conducted in the UAE in 2020 (Jarrar et al. 2022), participants were randomized into three groups: a control group, a WhatsApp intervention group, and an electronic brochures group. The aim was to evaluate the effectiveness of digital platforms in enhancing population knowledge regarding salt reduction (Jarrar et al. 2022). Results indicated that intervention through WhatsApp led to a noteworthy decrease in salt intake compared to the other two groups. Moreover, this reduction was accompanied by a decrease of 10% among participants who exceeded recommended salt intake levels in the WhatsApp intervention group (Jarrar et al. 2022).

This finding may serve as a valuable lesson for other Middle Eastern countries, suggesting that adopting similar digital intervention strategies could effectively increase population awareness and knowledge regarding salt reduction.

## 8 Economic Impact of Excess Salt Intake

When choosing a salt reduction policy, cost‐effectiveness is a crucial factor to consider. The healthcare systems across the world are under more pressure due to the aging population and advanced technologies, which result in increased demand (Mason et al. 2014). Therefore, governments should weigh both the expenses and advantages of all policies implemented. Consequently, in low‐ and middle‐income nations, where there are constraints on resources, it becomes increasingly crucial (Ayoub Al Jawaldeh et al. 2018). Hence, comprehending the economic consequences of salt reduction policies is vital for assessing their cost‐effectiveness, feasibility, and long‐term viability in public health interventions aimed at decreasing salt intake in the Middle East regions (Ayoub Al Jawaldeh et al. 2018).

### 8.1 Costs of Salt‐Related Health Issues

Salt‐related health issues present significant challenges to individual well‐being and impose substantial economic burdens on healthcare systems, particularly in Middle Eastern regions (Mason et al. 2014). It is estimated that over half of the yearly fatalities equivalent to 2.2 million fatalities and 60% of the disease impact in the Eastern Mediterranean area stem from NCDs (Ayoub Al Jawaldeh et al. 2018). NCDs such as CVDs, hypertension, and strokes, which are strongly associated with excessive salt intake, contribute to rising healthcare expenditures and productivity losses in the region (Cheikh Ismail et al. 2019). Hospitalization costs for cardiovascular events such as strokes and myocardial infarctions account for a significant portion of healthcare spending (Cheikh Ismail et al. 2019). Evidence increasingly suggests that it is economically feasible to implement salt reduction policies, particularly in high‐income countries (Cheikh Ismail et al. 2019). For instance, recent research undertaken in KSA revealed that hospital admissions for ischemic heart disease attributed to high salt intake averaged $10,710 per patient from 2000 to 2012, and the leading cause of death had a mortality rate of 21.7% in 2012 (Balaha et al. 2023). Similarly, Tunisia showed that the annual cost of hospital admission for acute myocardial infarction was $14,273 per patient (Mason et al. 2014). Furthermore, the long‐term management and treatment of hypertension, including medication expenses and outpatient consultations, place a significant financial burden on both individuals and healthcare systems. Likewise, a research study conducted in the US aimed to investigate the potential effects of reducing daily dietary salt intake by 3 g. The study predicted that such a reduction could result in annual healthcare expenditure savings between $10 billion and $24 billion (Wang and Labarthe 2011). Additionally, it forecasted a significant reduction in the occurrence of CHD, stroke, and myocardial infarction (Wang and Labarthe 2011). In addition to direct healthcare costs, salt‐related health issues such as CVDs and hypertension contribute to increased absenteeism and productivity losses due to illness and disability (Meier et al. 2015; Ye et al. 2023). One of the studies that examined this association was carried out in the USA, where it highlighted the association between high sodium intake and elevated rates of work and school absenteeism due to illness, with figures indicating a 51% increase among adults and a significant 77% rise among children (Ye et al. 2023). Moreover, the premature mortality and disability resulting from salt‐related conditions further compound these productivity losses, posing significant challenges to economic development (Ye et al. 2023). Effective public health interventions targeting salt intake not only have the potential to reduce healthcare costs but also to enhance workforce health and productivity, thereby fostering sustainable economic growth in the long term (Ye et al. 2023). Research on this association is limited in the Middle East.

### 8.2 Cost‐Effectiveness of Salt Reduction Policies

Assessing the cost‐effectiveness of salt reduction policies is paramount in determining the most efficient strategies for improving public health outcomes and mitigating the economic burden of salt‐related health issues in the Middle East. Various policies are available to governments aiming to decrease dietary salt intake. This includes initiatives such as raising health awareness and labeling food packaging to indicate salt content, which serves to promote public awareness and motivate individuals to limit their salt consumption (Mason et al. 2014). Additionally, partnering with food manufacturers can facilitate the voluntary reformulation of processed foods or the implementation of mandatory regulations on salt content. Fiscal measures, such as taxing high‐salt products, also present a viable strategy (Ayoub Al Jawaldeh et al. 2018). A Tunisian study revealed the notable cost‐effectiveness of policies emphasizing reformulation and labeling, showcasing substantial expense‐saving advantages. Notably, the incremental costs per life‐year gained were estimated at $14,000 for labeling and reformulation measures, underscoring their efficacy in enhancing public health outcomes while efficiently utilizing resources (Mason et al. 2014). However, the health promotion policy in Tunisia demonstrated a higher incremental expense per life‐year gained, exceeding $150,000, signaling its comparatively lower cost‐effectiveness compared to alternative interventions (Mason et al. 2014). Similarly, in Syria, the analysis identified health promotion and labeling policies as expense‐saving measures, aligning with Tunisia's findings. Notably, policies involving reformulation initially sustained high costs (Mason et al. 2014). However, when integrated with health promotion and labeling efforts, they transitioned into cost‐saving initiatives, underscoring the importance of comprehensive policy approaches in enhancing cost‐effectiveness. Despite their initial expense, policies integrating reformulation exhibited low incremental costs per life‐year gained, falling below $5000, further emphasizing their economic viability (Mason et al. 2014). The findings in the Middle East were similar to those shown in a recent study conducted in the USA, which proposed that if the government collaborates with manufacturers to decrease salt levels in processed foods, it has the potential to produce an additional two million quality‐adjusted life years and achieve yearly savings of more than US$32 billion in healthcare expenses (Dehmer et al. 2020). Assessing the cost‐effectiveness of salt reduction policies is essential for guiding public health priorities in the Middle East. Regional analyses, such as those from Tunisia and Syria, highlight that while reformulation policies may initially involve high costs, their integration with health promotion and front‐of‐package labeling transitions them into cost‐saving initiatives. This comprehensive approach has demonstrated incremental costs per life‐year gained falling below $5000, emphasizing their strong economic viability and long‐term sustainability (Mason et al. 2014). Importantly, these findings align with global evidence, including a recent U.S. study showing that government collaboration with food manufacturers to reduce salt levels in processed foods could yield an additional two million quality‐adjusted life years and save over US$32 billion annually in healthcare costs (Dehmer et al. 2020). Together, these regional and international insights underscore that the most effective and economically sound strategies involve comprehensive policy packages that combine reformulation, labeling, and public awareness campaigns.

### 8.3 Healthcare Expenditure Savings

Reducing salt intake has the potential to generate substantial savings in healthcare expenditures by mitigating the burden of salt‐related health conditions. Research indicates that a modest reduction of 3 g in daily salt intake could potentially yield significant health benefits (Messerli et al. 2021). This includes an estimated annual decrease in newly diagnosed CHD cases ranging from 60,000 to 120,000, stroke cases from 32,000 to 66,000, and myocardial infarction occurrences from 54,000 to 99,000, along with a decline in overall mortality by 44,000–92,000 cases (Messerli et al. 2021). Moreover, this reduction is projected to lead to significant savings equivalent to 194,000–392,000 years of life adjusted for quality and an annual decrease in healthcare costs ranging from $10 billion to $24 billion (Messerli et al. 2021). These outcomes are indicative of the potential for reduced healthcare utilization and lower rates of hospital admissions. Another study conducted in the US reported that reducing the mean salt intake by 9.5% through collaborating with manufacturers could result in significant healthcare expenditure savings. Specifically, the study projected annual savings of over US$32 billion in medical costs and an additional gain of two million quality‐adjusted life years (Wang and Labarthe 2011). Similarly, in the Eastern EMR, an assessment was conducted in Palestine; simultaneous implementation of all three policies—labelling, health promotion, and reformulation policies for lowering salt intake—leading to an anticipated 30% reduction in salt intake could yield substantial benefits (Ayoub Al Jawaldeh et al. 2018). Specifically, it was projected to generate an estimated cost saving of $6,000,000 and 2682 years of life gained, consequently reducing the rate of hospital admission (Ayoub Al Jawaldeh et al. 2018).

## 9 Research Priorities and Program Evaluation

Monitoring and evaluating salt intake involves planning interventions tailored to the local context and working within program constraints. Different strategies have been employed, including assessing salt usage, encouraging food companies to produce less salty products, and educating people about healthy eating (Rosewarne et al. 2021; Nawal Alhamad et al. 2015; Almedawar et al. 2015; Sarrafzadegan 2023; Al‐Jawaldeh, Rayner, et al. 2020). In India, the salt reduction strategy was evaluated using qualitative methods such as in‐depth interviews and discussions through focus groups with stakeholders (Gupta et al. 2018).

To monitor and evaluate salt intake levels, various methods are employed. Community health volunteers conduct home visits, and periodic assessments are carried out at educational institutions and community events. Salinity test kits are utilized for food and urine samples, ensuring accessible and cost‐effective monitoring (Opasanant and Sukwong 2023; Santos et al. 2021). These methods ensure accessible and cost‐effective monitoring of salt consumption in populations.

Other methods for monitoring and evaluating salt intake levels include the use of dietary self‐reporting instruments such as food records or 24‐h dietary recalls, spot urine samples, and the collection of 24‐h urine (Almedawar et al. 2015; Soh et al. 2022). Although the gold standard is collecting 24‐h urine, spot urine samples are more convenient, especially in low‐income countries. However, they may have limited validity across different ethnic groups (Soh et al. 2022). Additionally, methods for monitoring and evaluating salt intake levels include menu labeling, setting targets for salt reduction, and reformulating recipes. These methods aim to provide consumers with information to make healthier choices and encourage restaurants to offer low‐salt options. However, the effectiveness of these methods varies, requiring ongoing evaluation and improvement (Ding et al. 2020; Almedawar et al. 2015; Nawal Alhamad et al. 2015). In terms of monitoring, nearly all Middle Eastern countries, except Palestine, have incorporated mechanisms into their national salt reduction strategies, although the scope and rigor of these efforts vary. Iran, Jordan, Lebanon, and Kuwait have employed population‐based dietary intake surveys, such as 24‐h recalls or food frequency questionnaires, to estimate sodium consumption (Nasreddine et al. 2014; Al‐Jawaldeh et al. 2021). Gold‐standard biomarker monitoring through 24‐h urinary sodium excretion studies has been undertaken in Iran, Lebanon, and Saudi Arabia, providing more accurate assessments of intake levels. Food composition and labeling surveillance is prominent in Turkey, Saudi Arabia, and the UAE, where sodium content in key foods such as bread, processed meats, and snacks is periodically tested to evaluate compliance with reformulation targets (Musaiger et al. 2011; Al‐Jawaldeh et al. 2021). In addition, Saudi Arabia and Oman have strengthened industry compliance monitoring by routinely assessing sodium levels in reformulated products against national standards (Alhamad et al. 2015). Broader nutrition monitoring frameworks are also evident: Qatar and Bahrain have embedded sodium reduction into school and workplace health initiatives, conducting regular evaluations of food procurement and menu offerings (Al‐Jawaldeh et al. 2021). Finally, Egypt and Iran have integrated sodium intake surveillance into their national non‐communicable disease monitoring systems, aligned with the WHO STEPwise approach, ensuring that salt reduction progress is linked to overall NCD prevention efforts (World Health Organization. Regional Office for the Eastern Mediterranean 2019). Collectively, these strategies reflect a combination of biomarker surveillance, food environment monitoring, and integration into NCD frameworks, which together provide a robust basis for evaluating progress in sodium reduction.

Future analysis should consider the long‐term health care costs postponed by salt reduction policies, which may include productivity gains, changes in workforce participation, and tax revenue (Mason et al. 2014). Future interventions, such as establishing national task forces, reducing salt in bread, and monitoring sodium intake, are crucial. Interestingly, with the exception of Palestine, all 13 Middle Eastern countries are implementing multifaceted salt reduction interventions, combining two or more strategies. Taxation is the least common strategy, with only Qatar planning to implement it (Nawal Alhamad et al. 2015; Al‐Jawaldeh, Rayner, et al. 2020). Reformulation is the most common initiative, followed by user education, initiatives in given settings, and FOPL (Al‐Jawaldeh, Rayner, et al. 2020). Additional research should evaluate salt reduction approaches such as a salt tax and consider broader health and economic impacts (Mason et al. 2014; Leopold Ndemnge et al. 2020).

## 10 Recommendations for Bridging the Data Gaps in Sodium Consumption

One of the key limitations identified in this review is the scarcity of reliable and comprehensive data on sodium consumption across Middle Eastern countries. To address this, several measures are recommended:

1. National Nutrition Surveys: Governments should prioritize periodic, nationally representative dietary surveys that include accurate sodium intake assessment through standardized 24‐h urinary sodium measurements and validated dietary recall tools.
2. Use of Digital Platforms: Low‐cost, scalable technologies such as mobile health applications and WhatsApp‐based interventions can be employed for real‐time dietary monitoring and community engagement. These platforms may also support consumer education campaigns while generating valuable population‐level data.
3. Cross‐Country Surveillance Systems: Establishing a regional sodium surveillance framework, coordinated by WHO EMRO or regional health authorities, would allow harmonized methodologies, facilitate inter‐country comparisons, and track progress toward WHO salt reduction targets.
4. Integration with Existing Health Programs: Sodium monitoring should be embedded within broader non‐communicable disease (NCD) surveillance systems to ensure sustainability and efficient use of resources.
5. Capacity Building: Training programs for nutritionists, epidemiologists, and laboratory personnel are essential to strengthen national capacity for sodium assessment and data analysis.
6. Overcome industry resistance: Enforcing regional regulations on imported food items would ensure that external supply chains adhere to national sodium standards, thereby reducing the uncontrolled entry of high‐salt products.
7. Establishing inter‐ministerial task forces, including health, trade, education, and agriculture ministries, to promote cross‐sector collaboration and provide the sustained leadership necessary to implement and monitor salt reduction initiatives effectively.

## 11 Future Directions for Salt Reduction Consumption in the Middle East

Future directions for salt reduction policy in the Middle East include several key areas aimed at sustaining and enhancing efforts to reduce excessive salt intake and its related health risks. Regular review and update of salt reduction policies in the Middle East is crucial to ensure their continued effectiveness and relevance in meeting the changing dietary habits and health needs of the region (Hyseni et al. 2017). This will enhance the efforts toward reducing salt intake in the Middle East. The process may involve expanding policy coverage to include additional food categories like snacks, processed meats, and condiments, as well as enforcing stricter regulations on salt content in processed foods. Available studies showed that bread was most focused on and targeted by some Middle Eastern countries such as Bahrain, Qatar, and Palestine (Ayoub Al Jawaldeh et al. 2018). This assumes that governmental regulations would mainly apply to local bread manufacturers, which seems likely because locally produced bread products hold a significant market share in each respective country (Ayoub Al Jawaldeh et al. 2018). Therefore, it is essential to expand the scope of government regulations to include multinational food producers importing products into the region to ensure comprehensive coverage of all food products that contribute to salt intake. This can be achieved by promoting collaboration between local and international stakeholders to establish uniform guidelines and standards for salt content in food products across borders (Webster et al. 2014). Furthermore, implementing effective monitoring and enforcement mechanisms would be vital to ensure compliance with regulations and address any potential loopholes in the supply chain (Webster et al. 2014). Adopting a comprehensive approach that includes both locally sourced and imported food products can help governments regulate salt content more effectively and promote healthier dietary habits among the population (Webster et al. 2014). To achieve this, setting lower sodium targets for food manufacturers and providing clear guidelines for compliance is crucial. Moreover, improving enforcement mechanisms, such as regular inspections and penalties for non‐compliance by food manufacturers and restaurants, is also important to ensure that salt reduction policies are being followed throughout the food industry (van der Westhuizen et al. 2023). In addition, investing in research initiatives and vigorous monitoring mechanisms is vital for advancing salt reduction efforts. Research should focus on understanding the relationship between salt consumption and cultural dietary habits, as well as its impact on health outcomes unique to the region, while surveillance systems can track progress and evaluate policy effectiveness. Advocating for salt reduction policies at regional and national levels is crucial for raising awareness and gathering political support (Kantachuvesiri et al. 2024). By highlighting the economic and health benefits of reducing salt, policymakers can be encouraged to prioritize and invest in salt reduction initiatives as part of broader public health strategies (Kantachuvesiri et al. 2024).

## 12 Conclusion

In summary, this review offers a detailed overview and examination of salt reduction policies in different Middle East regions, emphasizing the crucial need to reduce excessive salt intake for public health. It covers the analysis of currently implemented policies in the region, explores salt‐related health impacts, discusses implementation strategies, emphasizes the importance of stakeholder engagement, and acknowledges existing challenges. It is evident that although progress has been made in implementing policies to reduce salt consumption, significant challenges remain, including cultural factors, dietary habits, and limited research. Moving forward, policymakers must prioritize evidence‐based strategies to overcome barriers and sustain salt reduction efforts in the region. Additionally, further research is necessary to be done specifically in the Middle East to address gaps in knowledge and inform future policy development. Also, to offer a broader view of the national‐level impact of salt‐related policies. Ultimately, concerted collaboration and action among governments, healthcare professionals, Non‐Governmental Organizations, and the private sector will be essential for achieving successful reductions in salt intake levels and improving public health outcomes in the Middle East.

## Author Contributions

**Fatima al Zahra Yakti:** writing – review and editing (equal). **Fathima Sahar Faisal:** writing – review and editing (equal). **Juman Ali Yaghi:** writing – review and editing (equal). **Lana Basel Abusalah:** writing – review and editing (equal). **Syed Zamzam:** writing – review and editing (equal). **Reema Tayyem:** conceptualization (equal), writing – review and editing (equal). **Tahra ElObeid:** conceptualization (lead), writing – review and editing (equal).

## Disclosure

The authors have nothing to report.

## Ethics Statement

The authors have nothing to report.

## Conflicts of Interest

The authors declare no conflicts of interest.

## Data Availability

The data presented in this study is available on request from the corresponding authors.
